# Thymidylate Synthase as a Predictive Biomarker for Pemetrexed Response in NSCLC

**DOI:** 10.1155/2013/436409

**Published:** 2013-12-25

**Authors:** Ali A. Bukhari, Ranjit K. Goudar

**Affiliations:** ^1^Eastern Virginia Medical School, 700 West Olney Road Norfolk, Norfolk, VA 23507, USA; ^2^Virginia Oncology Associates, Eastern Virginia Medical School, 5900 Lake Wright Drive Norfolk, Norfolk, VA 23502, USA

## Abstract

In recent years, major strides in cancer research have made it possible to select personalized chemotherapy recommendations based on an individual patient's tumor biology. The prognostic and/or predictive ability of biomarkers seeks to tailor the use of targeted chemotherapy and can result in improved clinical outcomes with reduced toxicity. A proliferation of new technology and pharmacotherapeutics in the setting of current FDA Clinical Laboratory Improvement Amendment (CLIA) standards has resulted in a recent surge in direct-to-physician biomarker tests. However, in the absence of clinical validation, there is the concern that the biomarkers may be utilized prematurely, resulting in improper chemotherapy selection and patient harm. Thymidylate synthase (TS) has been marketed as a predictive biomarker for the use of pemetrexed in NSCLC. We will examine the evidence behind the use of TS as a predictive biomarker to predict response to pemetrexed in NSCLC. At this time, the evidence does not currently support using TS assays to guide chemotherapy selection outside of a clinical research protocol.

## 1. Introduction

This paper reviews the literature regarding thymidylate synthase as a predictive biomarker for pemetrexed response in NSCLC. It aims to identify the lack of clinical validation and the harms of direct-to-physician biomarkers assays and their use outside of a research protocol. It also covers the different modalities available for biomarker assays and the lack of standardization in TS quantification.

## 2. Background

Lung cancer remains one of the most frequently diagnosed cancers in the United States; in 2013, over 225,000 Americans will be diagnosed with lung cancer, including over 125,000 who will present with metastatic disease [1, 2]. Unfortunately, the 5-year survival of non-small-cell lung cancer (NSCLC) remains only 16%, with little improvement over the last 30 years. The ECOG 1594 trial demonstrated equal efficacy of several platinum-based doublets (PBDs), but the trial was published before the advent of pemetrexed chemotherapy [3]. Medical oncologists must select from several chemotherapy regimens for the initial treatment of EGFR wild-type, ALK rearrangement-negative advanced NSCLC, including pemetrexed. In this paper, we will examine the evidence behind the use of thymidylate synthase as a biomarker to predict response to pemetrexed in NSCLC.

Pemetrexed has shown efficacy in advanced non-small cell lung cancer (NSCLC) not only in combination with cisplatin as first-line therapy [4] but also as a single agent for second-line treatment [5] and single-agent maintenance [6]. It is a multitargeted agent whose metabolites inhibit three folate-dependent enzymes: thymidylate synthase (TS), dihydrofolate reductase (DHFR), and glycinamide ribonucleotide formyltransferase (GARFT) [7, 8]. TS inhibition is the most important function of pemetrexed since the drug is only a weak inhibitor of GARFT, and DHFR activity/tetrahydrofolate oxidation is dependent on TS activity [9]. Pemetrexed also has a favorable toxicity profile including less hematologic toxicity and neurotoxicity than other commonly used lung cancer regimens [5, 10]. It is this favorable toxicity profile that is so appealing about pemetrexed use, particularly if it could be selected preferentially as first-line therapy. This forms the basis of our utilization of TS as a predictive biomarker.

Recent advances in research have allowed for chemotherapy recommendations to be tailored specifically to an individual patient's tumor biology. So-called “directed-chemotherapy” is used with the goal of improving patient outcomes and avoiding the toxicity of ineffective chemotherapy. For example, pemetrexed has been shown to be somewhat more effective in nonsquamous NSCLC compared to squamous cell cancer, and now is only FDA-approved for nonsquamous histology [4]. In preclinical studies, higher tissue levels of thymidylate synthase appear to correlate with reduced sensitivity to pemetrexed [11, 12]. Additionally, squamous cell carcinomas tend to express higher levels of thymidylate synthase [13, 14] which may explain why nonsquamous cancers appear more sensitive to pemetrexed. This raises the possibility that lower levels of TS expression may identify a subset of squamous tumors with increased sensitivity to pemetrexed. However, optimal methods of measuring TS levels and the validity of this level as a predictive biomarker have not been widely accepted.

Prognostic biomarkers are laboratory or pathology tests that correlate with a patient's overall survival (OS) in relation to their disease, independent of the type of treatment received. On the other hand, predictive biomarkers are associated with response to a specific therapeutic agent. Some markers may be both prognostic and predictive. For example, high levels of ERCC1 and RRM1 expression are both *prognostic* of better survival for patients with NSLCLC when compared with patients with lower levels [15–18]. Low ERCC1 expression may be *predictive* of response to platinum chemotherapy, and low RRM1 expression may be *predictive* of response to gemcitabine chemotherapy [16, 19–21]. However, in a recent review of biomarker studies involving ERCC1 and RRM1, the authors found a consistent lack of survival benefit in both retrospective and prospective trial designs that aimed to utilize previous biomarker data for chemotherapeutic selection [22]. Moreover, Friboulet et al. recently failed to revalidate the predictive efficacy of ERCC1 in both a new cohort (Lung Adjuvant Cisplatin Evaluation, LACE) as well as a subset of patients from the International Adjuvant Lung Cancer Trial (IALT) [23]. The authors attribute the lack of findings to a lack of technology in detecting responsible ERCC1 isoforms and/or a change in the performance of the 8F1 antibody that initially validated and confirmed the predictive efficacy of ERCC1. This debate over the clinical utility of ERCC1 serves to inform the discussion of the utility of thymidylate synthase assays in guiding chemoselection.

With such variability and overlap between prognostic/predictive attributes among biomarkers, both qualities need to be assessed independently. Low levels of TS expression may be prognostic of improved overall survival in NSCLC [24–26]. Still, the use of TS as a predictive biomarker for efficacy of pemetrexed remains unclear. Before any claims can be made, the validity of any such assay must be established.

A test that demonstrates analytic validity shows precision and accuracy that are reproducible in comparison to a gold standard, while clinical validity means that a test has a proven association with a specific clinical endpoint (i.e., response to a specific drug, recurrence risk, toxicity, etc.). It is quickly evident that while being analytically valid is important, a biomarker should only be used in clinical practice after successful clinical validation. However, a proliferation of new technology and pharmacotherapeutics in the setting of current FDA Clinical Laboratory Improvement Amendment (CLIA) standards has resulted in a recent surge in direct-to-physician biomarker tests for cancer patients. Without rigorous clinical validation, there is the concern that unvalidated biomarkers may be utilized prematurely resulting in improper chemotherapy selection and patient harm [27].

In the literature, two potential methods to measure TS expression have been described. These include (1) a semiquantitative immunohistochemistry score (*H*-score) defined by the degree of staining intensity (i.e., 0 = none, 1 = low, 2 = moderate, and 3 = high) multiplied by the percentage of positive neoplastic cells (range 0–300) [28, 29] and (2) RT-PCR to measure mRNA expression. Both of these assays are now commercially available. Since the semiquantitative *H*-score carries a subjective component, one could argue that a quantitative method of analysis such as PCR is superior.

## 3. Clinical Data

### 3.1. Retrospective Trials

Several retrospective studies have examined the correlation between TS levels and patient outcomes, with conflicting results (Table 1). These studies were quite small and often used different methods for quantifying or evaluating TS expression. Scagliotti et al. presented initial biomarker study data [30] shortly following the publication of their original phase III NSCLC study comparing pemetrexed/cisplatin (PC) versus gemcitabine/cisplatin (GC) [4]. Only a small fraction of patients had tumor available for testing (232 of 1725 total patients). The authors observed “weak associations between TS expression and clinical outcomes” with a treatment effect favoring PC over GC (*P* = 0.014) in patients with low-TS levels as measured by mRNA.

Silva and Cole reported TS expression by *H*-score on 16 patients (chart-reviewed 430 with metastatic NSCLC, 28 received pemetrexed, 16 had tissues available for staining) [31]. TS overexpressers, defined as an *H*-score > 5, had a 20% response rate (2 responders versus 8 nonresponders). Conversely, TS-negative patients, defined as an *H*-score < or = 5, had a 50% response rate (3 responders versus 3 nonresponders). Of the original 430 charts reviewed, 75% noted a defined histologic subtype with adenocarcinoma “being the most common.” However, the histology of the 16 study individuals was not reported. The authors concluded that TS expression could be useful as a predictive biomarker for pemetrexed in NSCLC with the caveat that larger controlled studies were necessary.

Chen et al. had 42 specimens available for TS staining with corresponding patient outcome data [28]. TS expression, assessed by *H*-score, demonstrated positive outcomes for pemetrexed use in NSCLC with low-TS versus high-TS levels resulting in PFS 4.8 versus 3.4 months (*P* = 0.01). Low-TS versus high-TS expressers were categorized by an *H*-score cutoff value of 150 (selected by ROC curve for efficacy analysis). Additionally, there was a trend towards improved OS in all patients with low-TS expression, but the difference was not statistically significant (21.4 versus 10.0 months; *P* = 0.09). Clinical treatment responses were also monitored during this study following the Response Evaluation Criteria in Solid Tumors (RECIST) [32]. The authors categorized response rate (RR) as a percentage of patients achieving complete response (CR) or partial response (PR) and disease control rate (DCR) as a percentage of patients achieving CR or PR or stable disease (SD) as outlined in RECIST. Response rates of 23% versus 15% (*P* = 0.70) and DCR of 73% versus 45% (*P* = 0.12) were noticed for low-TS versus high-TS patients. Thus, although clinical response rates were not significant, there was a positive trend noted for low-TS patients. When limited to patients with adenocarcinoma histology (83% of samples), PFS in low-TS and hig-TS patients was 4.8 versus 3.8 months; *P* = 0.03 with OS also being significant at 21.4 versus 10.0; *P* = 0.03. No differences in PFS and OS were observed in the squamous cell carcinoma subset (17%).

Igawa et al. provided further positive results with TS-negative patients reporting improved ORR, PFS, and OS (16.1% versus 0.0%, *P* = 0.05; 5.8 versus 1.6 months, *P* = 0.03; 14.7 versus 8.6 months, *P* = 0.04, resp.) [33]. In this study, TS expression was determined solely by IHC staining intensity with low expression (no stain or weak 1+, “TS-negative”) accounting for 57% of cases and high expression (moderate 2+ or strong 3+, “TS-positive”) making up the remainder. During this single-arm trial, patients received pemetrexed monotherapy. Most importantly, however, is that over two-thirds of the patients in this study received pemetrexed as third-line, fourth-line, or further treatment whereas the utility of TS as a predictive biomarker would be most applicable to pemetrexed use in a first-line setting as previously discussed.

Sun et al. had 193 samples available for TS analysis in patients who received pemetrexed-based chemotherapy (80% monotherapy, 20% platinum-doublet) [34]. Using an *H*-score analysis, TS-negative patients were more likely to respond to pemetrexed-based therapy than TS-positive patients (33.7% versus 14.1%, *P* = 0.002) and had longer median PFS (4.1 versus 2.0 months, *P* = 0.001). Subset analysis of patients receiving monotherapy continued to report positive results with response rates for TS-negative 28.4% versus 12.0%, *P* = 0.013 and median PFS 3.0 versus 2.0 months, *P* = 0.016. However, subset analysis of pemetrexed-platinum patients (*n* = 44) did not approach statistical significance, possibly the result of low power.

Christoph et al. also reported positive results with *H*-score analysis recently with a Caucasian population [29]. 196 samples from patients with advanced NSCLC treated with pemetrexed-based chemotherapy were available for TS analysis. TS-negative patients demonstrated not only improved median PFS (5.6 versus 3.5 months, *P* = 0.0131) but also longer OS (22.5 versus 11.5 months, *P* = 0.0107). This study is important both for its population demographics as well as the marked improvement in OS. Moreover, the authors did not observe a histology-related association of TS, a possible result of only 6% of samples being nonadenocarcinoma NSCLC.

In contrast to the positive results reported above, Chang et al. at the Samsung Medical Center in Seoul, South Korea, found no significant correlations between TS expression and clinical outcomes [35]. PFS in low-TS versus high-TS was 2.4 versus 1.3 months; *P* = 0.407, and OS in low versus high-TS was 9.5 versus 6.7 months; *P* = 0.688. TS expression was only through IHC staining intensity (80% TS-negative and 20% TS-positive) without further calculation of an *H*-score. Based on previous studies, one would expect better overall outcomes in a sample with predominantly low-TS expression levels. Chang et al. acknowledged several limitations including heterogeneity in TS staining within tumors as well as the lack of a standardized scoring system, both of which could account for these unexpected results. Again, similar to Igawa et al., this study was conducted in patients receiving pemetrexed as third- or fourth-line treatment. Thereby, limiting the scope of the work.

Shimizu et al. utilized RT-PCR for TS expression quantification [36]. While the authors found that responders had statistically significant lower TS expression levels as opposed to nonresponders (*P* = 0.0142), they failed to provide evidence for utility in other clinical endpoints (PFS: 18.0 versus 13.3 weeks, *P* = 0.3001).

Li et al. set out not to quantify TS and its association with pemetrexed-response but to analyze genetic polymorphisms in the promoter enhancer region of the *TS* gene [37]. While previous studies failed to find an association between TS expression genotypes and pemetrexed efficacy in advanced NSCLC [38], the authors found that in their study median PFS was longer (6.8 versus 3.8 months, *P* = 0.036) for patients with the “low-expressing” polymorphisms (2R/2R, 2R/3C, or 3C/3C) as opposed to the “high-expressing” polymorphisms (2R/3G, 3C/3G, or 3G/3G). RT-PCR was utilized to detect polymorphisms on 45 samples from chemonaïve patients with advanced NSCLC. Unfortunately, no difference in OS was observed (10.3 versus 10.1 months, *P* = 0.638). Authors argued that the lack of positive OS data could be the result of a decreased sample size.

One significant drawback to these retrospective studies was that they relied on banked tumor specimens and corresponding clinical data that were only available for a subset of participants. The resulting small sample sizes were likely underpowered to detect the true effect. While such variability in these studies precludes us from reaching any strong conclusions, they are useful in generating hypotheses for prospective trials.

### 3.2. Prospective Trials

In a small prospective study, Bepler et al. reported that TS levels correlated with disease response rates, but the results were not statistically significant [39]. Bepler et al. utilized RT-PCR to quantify TS in 35 patients with resectable NSCLC receiving neoadjuvant gemcitabine/pemetrexed. In the low-TS group, 39% of patients had CR or PR, compared to 29% of patients with high TS. A trend towards negative correlation between TS expression and response was noted, but did not meet statistical significance. A negative correlation implies that lower TS levels would be associated with a better clinical response and vice versa. Additionally, no significant differences in overall or disease-free survival were noted between high- and low-expressing TS patients [39].

Recently, Nicolson et al. have demonstrated correlation between TS levels (measured by nuclear IHC, cytoplasmic IHC, and mRNA) and pemetrexed efficacy [40]. This single-arm, phase 2 trial reported 70 nonsquamous NSCLC stage IIIB or IV patients receiving induction pemetrexed/cisplatin followed by maintenance pemetrexed in nonprogressors. When nuclear TS expression was evaluated using the *H*-score, the median PFS in the low expression group was 7.1 months compared to 2.6 months in the high expression group (*P* = 0.0015). Similar trends were seen when TS expression was evaluated by cytoplasmic TS *H*-scores (*P* = 0.09) and RT-PCR (*P* = 0.05).

## 4. Discussion

Taken together, the studies mentioned above thoroughly describe positive outcomes for a variety of clinic endpoints in low-TS patients treated with pemetrexed. In fact, even the two retrospective studies (Chang et al. [35] and Shimizu et al. [36]) that did not reach significance demonstrated a trend towards improved outcomes. More importantly, Nicolson et al. [40] in a prospective trial demonstrated clear positive results through a variety of TS quantification modalities.

Early biomarker studies of thymidylate synthase included patients with various histologies, including squamous cell carcinoma. While it is possible that the lack of positive results in some TS expression studies is the result of pemetrexed efficacy being histology-driven as previously reported [41], histology may merely be a surrogate for TS expression levels. Sun et al. demonstrated positive results not only for TS-negative patients but also for adenocarcinoma-only patients treated with pemetrexed-based therapy (median PFS 2.9 versus 1.4 months, *P* = 0.001) [34]. Recently, Maus et al. analyzed 1457 NSCLC samples of either adenocarcinoma (AC) or squamous cell carcinoma (SCC) for mRNA expression levels of TS, ERCC1, and RRM1 [42]. Each of the three biomarkers had lower expression levels in AC versus SCC (*P* < 0.001). Initial studies that pointed to higher pemetrexed efficacy in AC versus SCC were perhaps the result of significant variability between TS expression levels [4, 5].

While SCCs in general may have higher TS expression than ACs, it is possible that there may be some low-TS SCCs. If the link between low TS and improved response to pemetrexed is validated, then TS expression might serve to identify additional populations of patients with SCC who may benefit from this chemotherapy. In that event, a search to identify the rate of TS overexpression in SCC tumors would be a useful next step.

Unfortunately, TS protein expression and mRNA levels may not be strongly correlated. Chen et al. described significant discordance between mRNA and protein expression in lung cancer, raising the concern that PCR may not accurately reflect tumor biology [43]. Conversely, Nicolson et al. reported that IHC as well as RT-PCR expression of TS inversely correlates with PFS, suggesting that TS protein expression and mRNA levels both track together [44]. Such findings were shown for TS in CRC and gastric tumors by Johnston et al. and could hold true for advanced NSCLC as well [45]. With such variability between various assays, it is important that future studies include both IHC as well as RT-PCR in order to identify the most relevant assay.

The Samsung Medical Center (Seoul, Korea) is currently enrolling patients in a phase 2 study evaluating the predictive value of TS expression in advanced NSCLC treated with pemetrexed/cisplatin versus gemcitabine/cisplatin (ClinicalTrials.gov Identifier: NCT01401192). Unfortunately, no interim data analysis was available at the time of publication of this paper. The authors anticipate preliminary results early in 2014 [46].

Liu et al. recently published a meta-analysis supporting the role of TS as a predictive biomarker for pemetrexed response in NSCLC [47]. In their study, they identified 8 recent (2008–2013) trials that when pooled showed a relative risk (RR) of 2.06 (1.44–2.96 95% CI) for higher TS expressers versus lower and PFS hazard ratio (HR) of 0.63 (0.52–0.76 95% CI) for lower TS expressers versus higher. Once again, these stories were varied in terms of TS quantification (IHC versus *H*-score versus RT-PCR), small in sample sizes, and primarily relied on retrospective studies with banked tissue samples. The authors advocate for the role of prospective trials prior to any formal recommendations.

Prior to any prospective trial, TS assays need to be quantified and analytically validated so that parameters delineating high- and low-TS expression values may be established to assist in further study. We recommend that TS expression should be evaluated in multidimensional approach including IHC with expanded *H*-score (IHC intensity × percent positive neoplastic cells × 100) as well as RT-PCR as illustrated by Nicolson et al. [40]. After comparison of the test characteristics of both IHC and PCR, the optimal assay may be selected. Commercial assays of both types currently exist that can be validated in research settings.

Additionally, Li et al. presented data that promotes the use of single nucleotide polymorphism (SNP) analysis in promoter region of the TS gene as a surrogate for TS expression quantification [37]. The previous study mentioned above by Smit et al. [38] which failed to show similar findings was perhaps the result of ethnic variation given that the study was conducted in The Netherlands as opposed to China in the case of Li et al. The further development of this research will not only provide an alternative means for TS evaluation but also aid in the cost effectiveness for clinical utility.

A major limitation of current research is that most studies described above are retrospective, relying exclusively upon banked tissue specimens. Most of these studies utilized pemetrexed second- or third line. We need to consider the up- and downregulation of enzymes through exposure to multiple chemotherapy agents, as this undoubtedly alters tumor biology [48]. Thus, trials utilizing pemetrexed as first-line therapy would not only serve the purpose of preventing altered tumor biology but also validate our aforementioned goal of minimizing toxicity through preferential selection of pemetrexed in low-TS patients. As such, while the described trials provided significant evidence in favor of TS as a predictive biomarker, they should be viewed with the understanding that retrospective trials are primarily hypothesis generating and prospective trials would open the door for more accepted use.

Prospective trials in nonsquamous histology should be conducted randomizing patients into a 2 × 2 model involving high-/low-TS levels and pemetrexed/cisplatin versus another accepted platinum-based doublet. Such stratification by TS expression and randomization to treatment regimen would greatly enhance the quality of research currently available. Clinical endpoints should include measurement of PFS, OS, and ORR. The availability of all three major clinical endpoints would prove useful in evaluating the efficacy of TS as a predictive biomarker. As pemetrexed is currently only FDA-approved in the United States for nonsquamous histology, predictive efficacy should be established within that subtype before we can consider its potential use for a subset of SCC.

Ultimately, further prospective trials are necessary to help clarify the utility of TS quantification in guiding selection of pemetrexed as first-line chemotherapy for NSCLC. If validated, such a measure would prove useful to both patients and physicians by providing evidence to help guide the selection of first-line treatment in advanced NSCLC. Until the clinical validity of TS as a predictive biomarker is firmly established, the evidence does not support using TS assays to guide chemotherapy selection outside of a clinical research protocol at this time.

## Figures and Tables

**Table 1:** Retrospective studies evaluating TS as predictive biomarker for pemetrexed in NSCLC.

Authors	Method for TS quantification	Number of participants (*n*)	Histology	Results			
					TS-negative	TS-positive	*P* value
Silva and Cole, 2011 [31]	*H*-score (IHC intensity × % pos. neoplastic cells) [28]	16	Adenocarcinoma—75%	ORR	50%	20%	
Chen et al., 2011 [28]	*H*-score	42	Adenocarcinoma—83.8% Squamous cell carcinoma—16.7%	All patients			
				**PFS**	**4.8**	**3.4**	**0.01**
				OS	21.4	10.0	0.09
				Adenocarcinoma-only patients			
				**PFS**	**4.8**	**3.8**	**0.03**
				**OS**	**21.4**	**10.0**	**0.03**
Igawa et al., 2012 [33]	IHC Intensity	54	Adenocarcinoma—96.2% Other nonsquamous NSCLC—3.8%	**ORR**	**16.1%**	**0.0%**	**0.05**
				**PFS**	**5.8**	**1.6**	**0.03**
				**OS**	**14.7**	**8.6**	**0.04**
Sun et al., 2011 [34]	*H*-score	193	Adenocarcinoma—89% Other nonsquamous NSCLC—11%	**ORR**	**33.7%**	**14.1%**	**0.002**
				**PFS**	**4.1**	**2.0**	**0.001**
Christoph et al., 2013 [29]	*H*-score	196	Adenocarcinoma—75% Squamous cell carcinoma—5% Other NSCLC—20%	**PFS**	**5.6**	**3.5**	**0.0131**
				**OS**	**22.5**	**11.5**	**0.0107**
Chang et al., 2010 [35]	IHC intensity	55	Nonsquamous Cell Carcinoma—87% Squamous cell carcinoma—13%	PFS	2.4	1.3	0.407
				OS	9.5	6.7	0.688
Shimizu et al., 2012 [36]	RT-PCR	50	Unavailable	PFS	18.0 weeks	13.3 weeks	0.3001
Li et al., 2013 [37]	SNPs, RT-PCR	45	Adenocarcinoma—100%	**PFS**	**6.8**	**3.8**	**0.036**
				OS	10.3	10.1	0.638

ORR: overall response rate, %; PFS: progression free survival, months unless indicated; OS: overall survival, months unless indicated.

Bolded values are for *P* values < or = 0.05.
